# Study on Multi-Layer Filling Treatment of Extra-Large Goaf and Its Underground Application

**DOI:** 10.3390/ma15165680

**Published:** 2022-08-18

**Authors:** Huazhe Jiao, Wenbo Yang, Huiming Shen, Yingjie Yang, Juanhong Liu

**Affiliations:** 1 School of Civil Engineering, Henan Polytechnic University, Jiaozuo 454000, China; 2 School of Civil and Resource Engineering, University of Science and Technology Beijing, Beijing 100083, China

**Keywords:** large goaf, stratified cemented backfill, particle size analysis, strength reduction, grain flow

## Abstract

At present, the many domestic, large mined-out areas caused by single filling ability of the slurry flow state, thin layer flow and hardening after filling in multilayer structure generally need to finish filling for many times, because after a filling experience shows that filling body in the last solidification of flow, this leads to a lower one side of the roof, and far distance part of the filling body cannot pick up top. The determination of backfill strength is the key problem of the cemented backfill method, and it is affected by many factors. Therefore, through theoretical calculation, laboratory testing and numerical simulation methods, combined with the field filling process, this paper has verified the flow accumulation and stratification characteristics of stope layered filling slurry. When the slurry concentration is 60–73%, the slope increases exponentially from 2.5° to 8°. It is revealed that the delamination and meshing state are the key factors to determine the overall strength of large-scale stope filling through the testing of particle size distribution in interlayer and flow direction. The reduction effect of the number and Angle of structural weak surface formed by layering and filling on strength is revealed: cement–sand ratio 1:12, concentration 68%, standard curing R28 > 1.81 MPa. The strength reduction coefficient is 61.31% and 92.96% when the number of layers is 1–4. The higher the number of layers, the greater is the reduction coefficient, and when the stratification angle increases by 2, the strength of backfill decreases by 20–30%. The verification of stope filling coring shows that the in situ strength reaches 2.42 MPa, which is 0.61 MPa higher than the standard curing strength, with an increase of 33.7%. When the depth is from 1 m to 5 m, the strength increases from 2.26 MPa to 2.69 MPa, with an increase rate of 18.2%. Finally, through the research and application of the comprehensive technology of mining and filling coordination under the complex goaf group, the residual ore resources of Xianglushan tungsten mine are effectively recovered, the volume of goaf is significantly reduced, and the safety of goaf is improved.

## 1. Introduction

The volume of underground goaf in China is about 1.3 billion m^3^, and many mines in China have large exposed area and high-density goaf groups, which lead to the deterioration of mining environment, great threat to underground personnel and equipment, and a large amount of economic losses to mines. For the goaf with a large area to be filled, the following problems generally exist in the filling process: First, the paste filling flow rate that has been built in China is generally 80–100 m^3^/h, and the single filling capacity is 300–800 m^3^. When the area to be filled is larger than 300–500 m^2^, the height of a single filling is less than 1 m, resulting in a thin layer flow of slurry and a multilayer structure of the hardened filling body. Second, the stope generally needs several filling operations to fill, and the later filling slurry flows on the surface of the previously consolidated filling body. After the solidification of the slurry, the filling body Angle makes the interior of the stope look like an inclined plane. The higher position is close to the roof, while the lower side is far from the roof, leading to part of the filling body unable to be connected to the roof. Thirdly, the particle distribution affects the strength of backfill in the process of paste flow consolidation. Paste with high concentration and uniform properties can be regarded as non-sedimentation fluid, but it is still possible to produce particle size classification in the actual filling process [1].

For these problems, scholars at home and abroad have done a deal of research in recent years. As for the influence of stratified structure on the mechanical properties and failure mode of backfill, Wang Jie et al. prepared different backfill samples under the conditions of 1:4 cement–sand ratio, 75% slurry concentration and 1, 2 and 3 backfill layers, respectively, and carried out triaxial cyclic loading and unloading tests. The results showed that with the increase of filling times, the peak strength of backfill decreases as a polynomial function, and the peak strain increases as an exponential function [2]. Jie Wang et al. established a damage constitutive model by studying the mechanical properties of backfill with different interlayer ratios and heights [3]. Shuai Cao et al., studied the influence of filling interval time on the uniaxial compressive strength of cemented tailings backfill [4]. As for the slope problem caused by filling, Sadegh Javadi et al. discussed three areas of flow path in the study, namely, the first section is the subduction pool area, and energy dissipation occurs in the process of mortar flow from pipe to open channel flow. The second section is the open channel flow zone where mortar leaves the subduction pool in the form of open channel flow. The third section is the sedimentary sector, and the mortar is fan-shaped [5]. Morris et al. established the mechanical model in the flow process of open channel and carried out experimental verification, and proposed the slope Angle prediction model of unmacabellar slurry [6,7]. Thompson et al. studied the mechanical behavior of Newtonian and non-Newtonian fluids in the slope flow process respectively. In addition, a combination of theoretical analysis and numerical simulation was used to predict the relevant angles [8,9].

**Figure 1:**
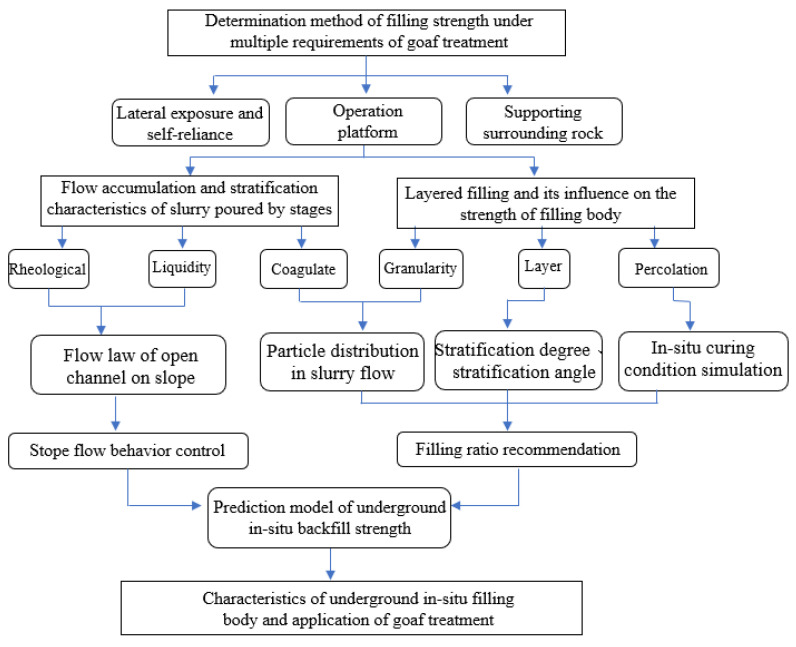
Work flow chart.

To sum up, for the common mined-out areas with small area, the filling method of high strength filling materials in the upper and lower parts of the mined-out areas and low strength filling materials in the middle part can be used, which can effectively reduce the cost. However, the previous filling method is not suitable for the extra-large mined-out areas, because the large-area and large-volume mined-out areas are affected by the filling speed, and they must be filled for many times before they can be completed, which results in the existence of structural planes among the filling bodies for many times, which has a significant impact on the overall strength of the filling bodies. The existing strength design standard of backfill only considers the strength of the material itself, but ignores the structural and mechanical characteristics between the backfill and surrounding rock [10], especially the arch effect inside the backfill, resulting in the strength design standard of backfill much higher than similar foreign mines [11,12]. Therefore, this paper carried out several layered simulation tests of backfill based on previous studies to detect the strength of backfill, so that the strength of each layer of backfill can meet the filling standard. Study the flow diffusion behavior of filling slurry in stopelike open channel, carry out small-scale test sampling to detect the particle distribution law of slurry slope flow process, analyze the influence mechanism of particle distribution on flow and solidification, and obtain the relationship between slope thickness Angle and concentration. To reveal the mechanism of flow consolidation behavior and its influence on backfill delamination, PFC discrete element was used to simulate the failure process of multilayer backfill blocks to reveal the influence mechanism of backfill times on backfill strength reduction. Finally, downhole test block maintenance and in situ drilling core strength test were carried out to compare the promoting effect of downhole test block maintenance and in situ curing conditions of backfill on strength. The application of filling technology to treat underground mined-out area creates conditions for residual ore mining. The workflow is shown in Figure 1.

## 2. Materials and Methods

### 2.1. Material

**Figure 2:**
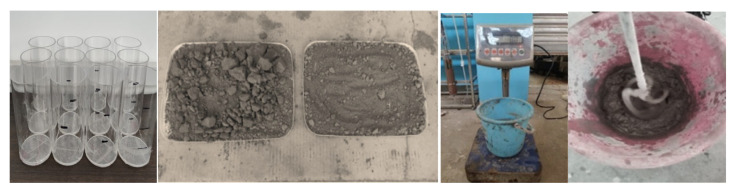
Preparation of experimental filling material.

The whole tailings of Xianglushan Tungsten mine were used as raw materials, 42.5 R ordinary Portland cement was used as cementing agent, and the experimental water was taken from laboratory tap water. The mold adopts a high transparent acrylic tube with a height of 100 mm and an inner diameter of 50 mm. The experimental filling body is prepared as shown in Figure 2.

### 2.2. Research Methods

#### 2.2.1. Slope Flow and Slope Angle Test

**Figure 3:**
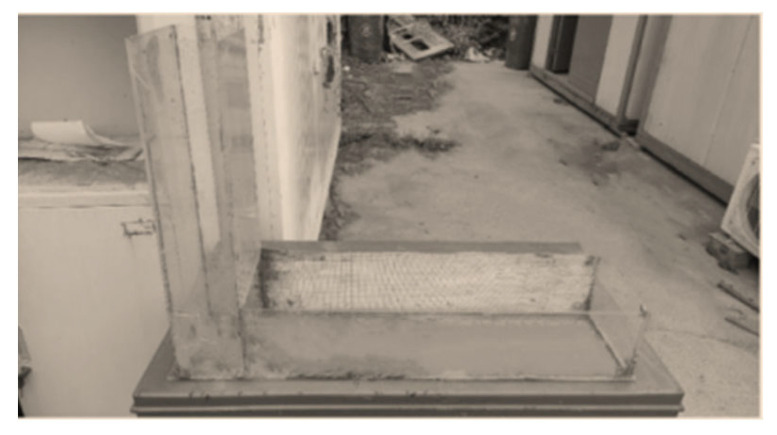
L chute.

**Figure 4:**
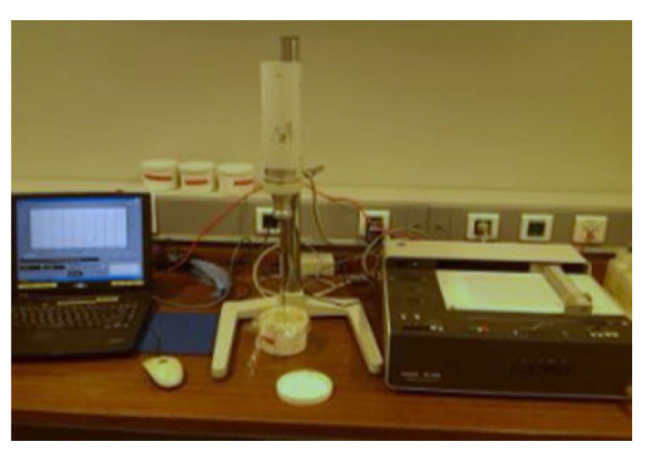
Rheometer detection.

Field tailings and cementing powder were used to carry out slope flow test of L-shaped flow groove with slurry of 66%, 68%, 70% and 72% concentration, as shown in Figure 3. The propeller rotor rheometer was used to detect the yield stress of slurry, the instrument is RS-CC rheometer produced by Brookfield Company, New York, NY, USA. as shown in Figure 4.

#### 2.2.2. Particle Distribution of Paste Filling Slurry Stacked in Layers

The particle size distribution along the flow direction affects the local strength of backfill, and the particle size distribution along the vertical direction is the direct cause of the structural weak surface of multilayer backfill [13,14,15]. Therefore, the particle size distribution characteristics restrict the development of backfill strength.

**Figure 5:**
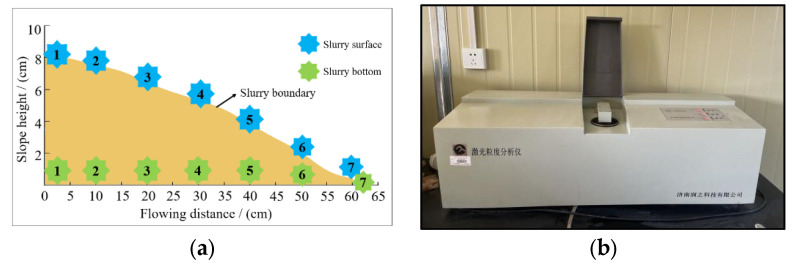
(**a**) Sampling location; (**b**) Laser particle size analysis.

After the completion of filling, the slurry will stop flowing, and samples will be taken at the same flow distance between the slurry surface and bottom surface along the flow distance direction with the following inlet as the starting point and 10 cm apart. The particle size of the sample was measured by laser particle size analyzer. The instrument used in the experiment was Rise-2008 laser particle size analyzer developed by Runzhi Technology Company in Jinan, China. The particle size distribution characteristics in the horizontal and vertical directions were analyzed. The sampling location is shown in Figure 5.

#### 2.2.3. Preparation of Cemented Backfill Specimen

The filling body test blocks with cement–sand ratio of 1:12, mass concentrations of 66%, 68% and 70%, and layers of 1, 2, 3 and 4 layers were prepared. The curing age of the specimen was 28 days, and the constant temperature and humidity curing box was adopted for curing, with the temperature of 20 ± 5 °C and relative humidity of 95% ± 5% [16].

**Figure 6:**
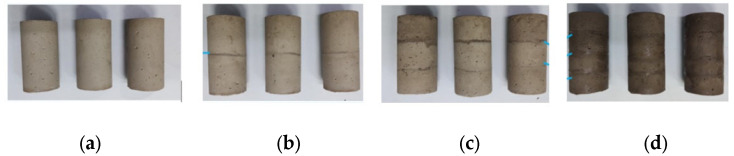
Delamination filling body test block: (**a**) One layer; (**b**) Two layers; (**c**) Three layers; (**d**) Four layers.

(1) Mixing and mixing: first mix cement, tailings and water according to the set proportion, and then put the slurry mixture under the mixer to mix evenly and reserve;
(2) Mold filling: first fill the bottom slurry according to the set height, then fill the second slurry according to the set height at an interval of 24 h, then fill the third slurry at an interval of 24 h, and finally fill the fourth slurry at an interval of 24 h to 100 mm height;
(3) Demolding curing: Put the filled specimen into the curing box, and take out demolding and continue curing until 28 d for reserve after curing for 3 d, as shown in Figure 6. The uniaxial compression test of filling body in different layers was carried out by gaW-2000 microcomputer controlled electro-hydraulic servo press.

## 3. Results

### 3.1. Flow Test

**Figure 7:**
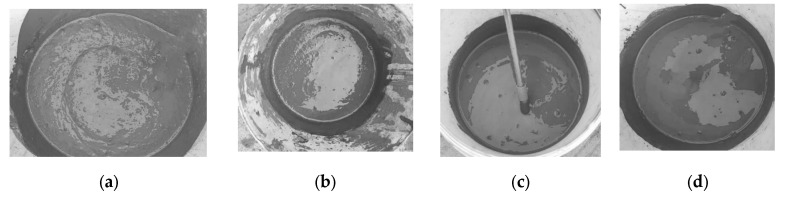
Preparation of all tailings paste: (**a**) 72%; (**b**) 70%; (**c**) 68%; (**d**) 66%.

**Figure 8:**
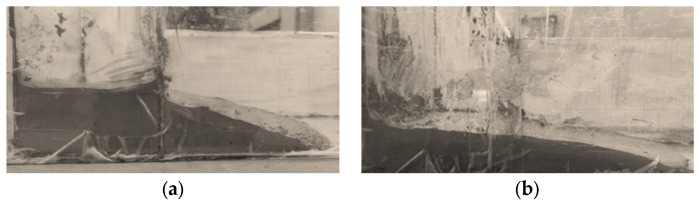

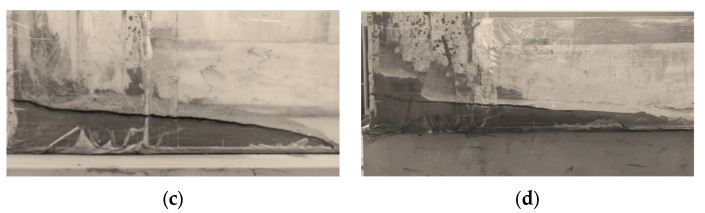
Effect of concentration on slope of tail mortar monolayer: (**a**) 72%; (**b**) 70%; (**c**) 68%; (**d**) 66%.

The preparation of all tailings paste is shown in Figure 7. After the preparation of the whole tailings paste sample, the single-layer flow test process of l-shaped flow groove is shown in Figure 8, and the test results are shown in Table 1.

**Figure 9:**
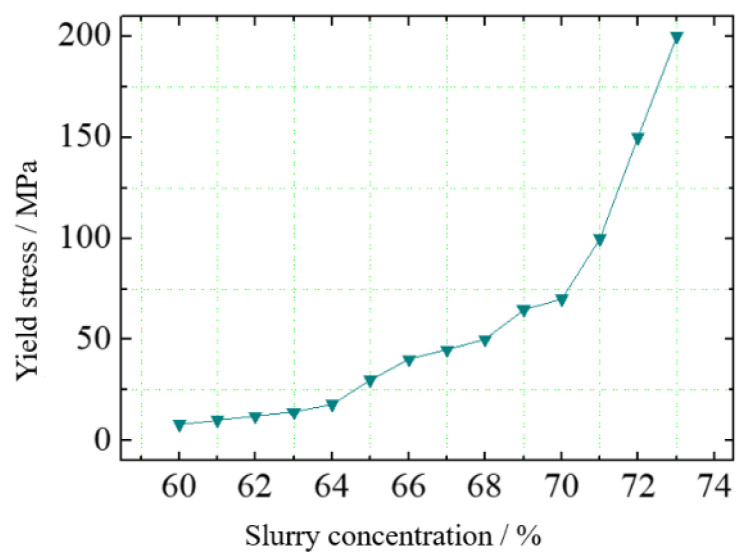
Prediction results of accumulation slope under the influence of concentration.

The rheometer test results of the whole tailings paste are shown in Figure 9. The yield stress of slurry increases exponentially with increasing concentration. When the concentration increases from 70% to 72%, the yield stress of slurry surges to more than 200 MPa.

**Figure 10:**
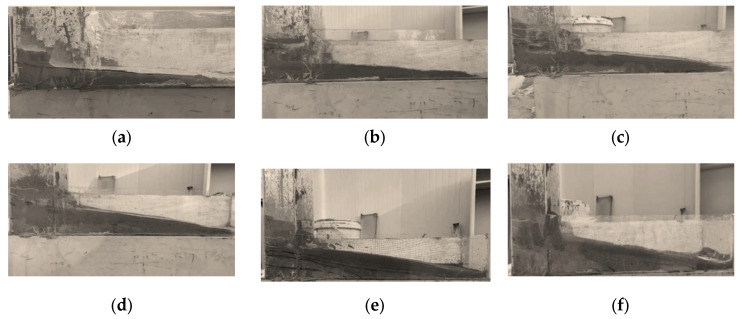
Flow accumulation profile of multilayer backfill body: (**a**) One layer; (**b**) Two layers; (**c**) Three layers; (**d**) Four layers; (**e**) Five layers; (**f**) Six layers.

According to the above test results, the full tailings paste slurry with 66% concentration is used to carry out multilayer backfill test. After each layer of filling slurry is dried, the next layer of filling slurry is injected into the feeding port, and the flow distance and Angle are detected after the flow stops. See Figure 10.

After the filling slurry enters the groove, the shear stress caused by gravity overcomes the yield stress of the slurry itself, resulting in the viscous flow of the slurry [17,18]. Due to the friction resistance between slurry and boundary and the cohesion of slurry, the energy loss causes the slurry to stop flowing and then the slurry enters the initial solidification stage. At the same time, when the shear stress caused by the material’s own gravity balances with the yield stress of the slurry itself, the slurry will be deposited, resulting in the deformation and remodeling of the surface profile of the slurry [19]. With the passage of time, the slurry water loss, slurry from liquid to solid, under the action of its own gravity, the consolidated body currently to produce effective stress, to provide support for the next layer of slurry flow.

**Figure 11:**
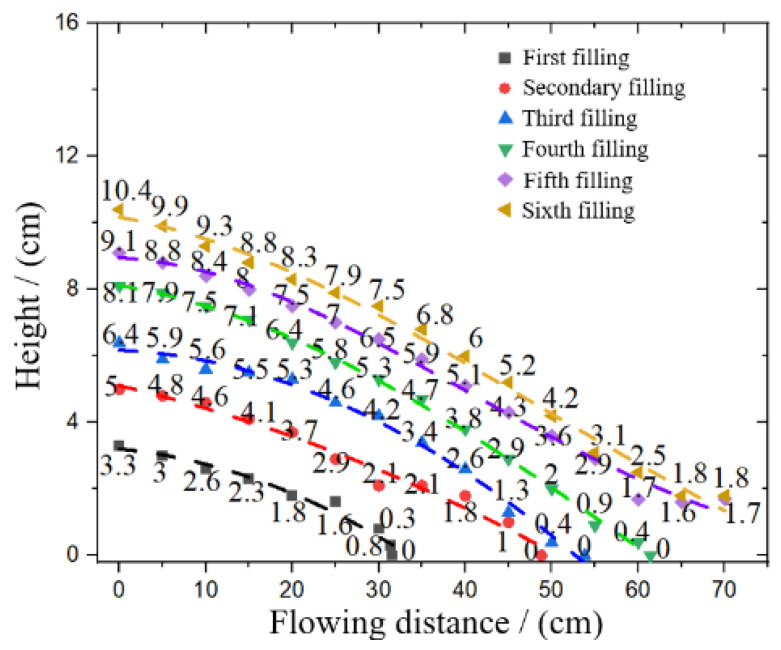
Flow accumulation slope fitting.

The slope curve was drawn, and the slope of each layer was nonlinear fitting. See Figure 11.

In the process of slurry flow, the yield stress of slurry itself decreases with the increase of flow spacing. The mechanical cause is shear thinning of the non-Newtonian fluid, and the microstructure is attributed to particle classification and flocculant rearrangement in the flow.

### 3.2. Particle Distribution of Paste in Slope Flow Process

A total of six slopes were formed in the multilayer stacking test with 66% concentration of filling slurry, and the representative bottom layer and the sixth layer were selected to carry out the study. Samples were taken on the bottom and surface of each backfill for particle size detection. The particle size distribution data along the flow direction are shown in Table 2 and Table 3.

It can be seen from the table that the particle size distribution characteristics of each layer flow slurry are as follows: the bottom particles are thicker than the surface particles are finer, and the farther the flow distance is, the finer the particles are. The particle size difference in the vertical direction is significantly greater than that in the flow direction.

(1) Along the flow direction

Happened in the process of liquid filling pulp layer segregation phenomenon, as a result of the coarse particle settling velocity relative to the fine particles is bigger, this makes the most of the coarse particle main deposit in the bottom of the fine particles are mainly concentrated on the surface, as the slurry to move forward, coarse particle precipitation stabilized, and the fine particles with the slurry flow gradually far end is pushed to the model. This results in stratification and segregation. In the flow direction, the particle size becomes fine with the increase of the flow distance. When it is close to the discharge point, the content of the particle size range of 75,300 μm is about 60%, and at the far end of stopping the flow, the particle size content of this range decreases to about 11–15%.

(2) Vertical direction

The bottom grain is significantly coarser in the vertical direction. After the flow of each layer stops, the particle size of the bottom surface concentrates in the range of 75–300 μm, and the content reaches more than 90%. The particle size of the surface concentrates in the range of 0.1–37 μm, and the content is generally more than 80%.

### 3.3. Uniaxial Compressive Strength

Uniaxial compressive strength is one of the most important mechanical parameters of backfill [20,21]. In this paper, uniaxial compression tests are carried out on horizontal and dip slicing backfill, and the results are shown in Table 4. With the increase of filling times, the uniaxial compressive strength of filling body specimens decreases. The compressive strength of the filling body increases with the increase of the concentration of the specimen with the same filling times.

In China, the strength value of complete backfill is usually used as a reference for theoretical calculation of backfill strength, whether for slicing mining, sublevel mining or stage subsequent mining [22,23]. By analyzing the data in Table 4, it is found that filling times have an obvious weakening effect on the strength of backfill. In other words, the more layers the filling body has, the smaller the overall compressive strength value of the filling body.

### 3.4. Strength Reduction Analysis of Layered Backfill Body

The concept of strength reduction coefficient of backfill is introduced [24], which is defined as:

(1) $$k=\frac{\sigma_{c}^{\prime }}{\sigma_{c}}$$

Type: $\sigma_{c}^{\prime }$ is the compressive strength (MPa) of the layered backfill; $\sigma_{c}$ is the compressive strength (MPa) of the complete backfill. The strength reduction coefficient is calculated based on the test data, and the results are shown in Table 5.

By analyzing Table 5, it is found that when the slurry concentration of filling material is constant, the strength reduction coefficient K decreases with the increase of filling times. It indicates that the weakening effect of compressive strength of backfill becomes more obvious with the increase of filling times. When the filling times were fixed, the K value showed an overall increasing trend with the increase of slurry concentration. When the filling body concentration is 66–70%, the corresponding K value is between 61.31–92.96%.

**Figure 12:**
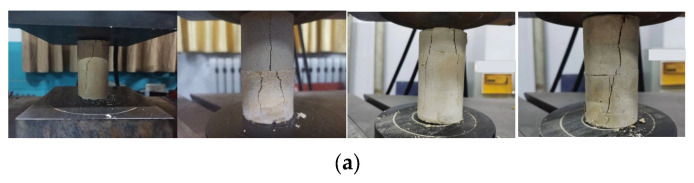

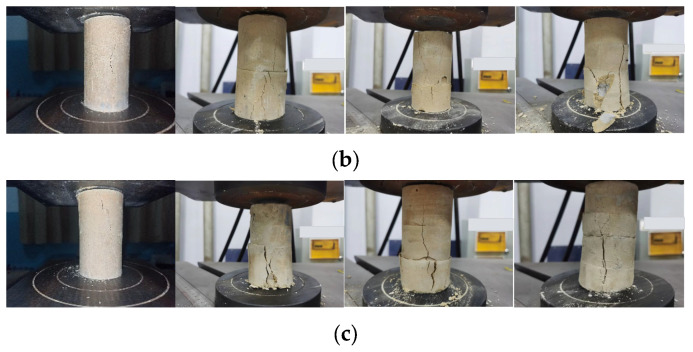
Failure modes of filling body specimens with different concentrations: (**a**) 66% filling body; (**b**) 68% filling body; (**c**) 70% filling body.

As shown in Figure 12, the failure modes of backfill specimens are mainly conjugate shear failure and tensile failure through the stratification plane. At the same time, by comparing and analyzing the failure forms of the specimens with filling times of 2, 3 and 4, it is not difficult to find that after the completion of the loading test, the specimens with failed backfill appear different degrees of separation and dislocation at the level. Due to the formation of low-strength interlayer between layers during interval filling, it is easy to be damaged during loading test, thus reducing the overall bearing capacity of the filling body.

### 3.5. Influence of Delamination Angle on Strength Characteristics of Backfill

**Figure 13:**
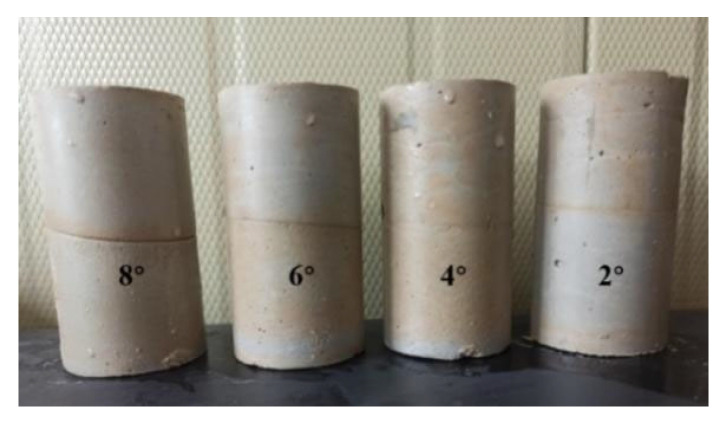
Preparation of dip Angle layered cemented backfill specimen.

The Angle between different backfill layers was taken as the main research object to influence the strength of cemented backfill. The ratio of slurry to cement sand was set as 1:12, the four concentrations were 64%, 66%, 68% and 70%, and the Angle between the layers of cemented backfill was set as 2°, 4°, 6° and 8°. When preparing specimens with different angles between layers, the filling time interval was set as 24 h, and the design curing age was set as 28 d. The strength of the layered backfill was studied. The production of cemented backfill samples with different angles between backfill layers is shown in Figure 13.

**Figure 14:**
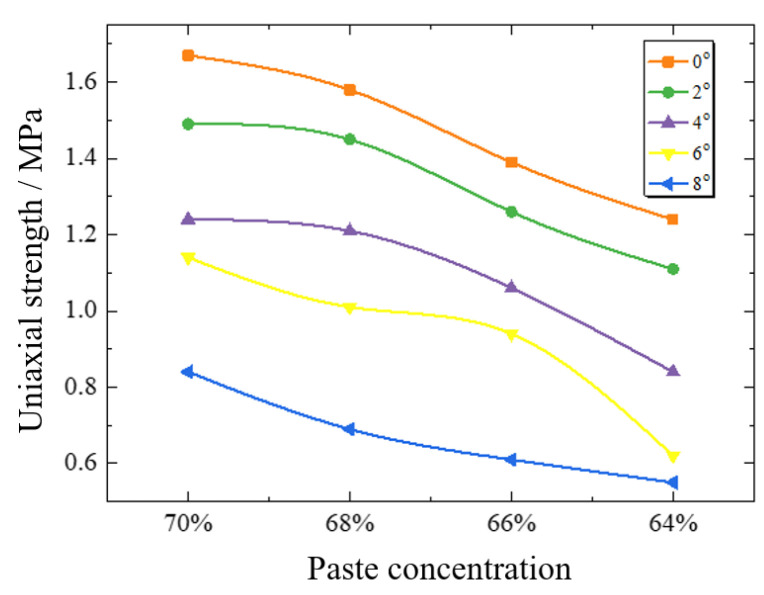
Strength of backfill under the influence of accumulation Angle.

The strength of backfill under the influence of stacking angle is shown in Figure 14. When the mass concentration is 64%, the uniaxial strength decreases by 55.46% from 1.24 MPa to 0.55 MPa as the delamination Angle increases from 0° to 8°. When the mass concentration is 66%, the uniaxial strength decreases by 56.22% from 1.39 MPa to 0.61 MPa with the increase of the delamination Angle. When the mass concentration was 68%, the uniaxial strength decreased by 56.22% from 1.58 MPa to 0.69 MPa. When the mass concentration was 70%, the uniaxial strength decreased by 49.44% from 1.67 MPa to 0.84 MPa. The higher the slurry concentration of backfill, the smaller the reduction coefficient of backfill strength.

As can be seen from Table 6, the uniaxial strength reduction ratio of backfill is about 89–91% at 2°, 67–74% at 4°, 50–68% at 6°, and 43–50% at 8°. When the delamination Angle increases by 2°, the reduction rate of backfill strength is maintained at 20–30% on average. The greater the delamination Angle, the greater the intensity reduction factor.

**Figure 15:**
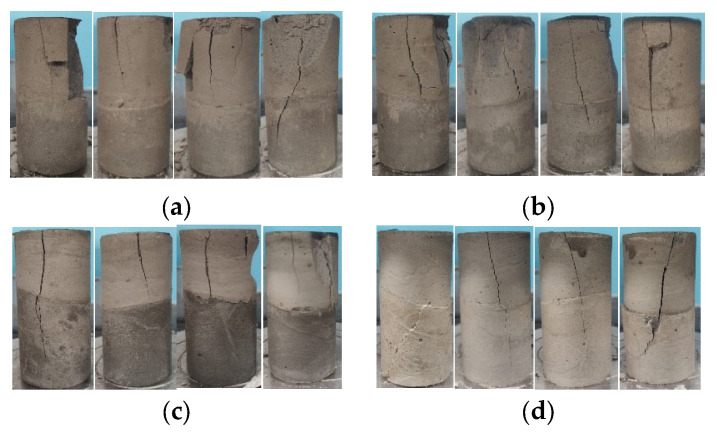
Failure mode of dip slicing backfill body: (**a**) 64%: 2° 4° 6° 8°; (**b**) 66%: 2° 4° 6° 8°; (**c**) 68%: 2° 4° 6° 8°; (**d**) 70%: 2° 4° 6° 8°.

The inclination degree of backfill has a significant impact on the strength of backfill samples [25,26]. Figure 15 shows the failure mode of the backfill specimen when the Angle of the backfill surface exists, which is manifested in the form of penetrating tensile failure and semi-penetrating shear failure parallel to the loading direction. The specimen of backfill body exhibits tensile failure, but also exhibits a shear failure trend along the backfill surface. The crack forms are the cracks at a certain Angle to the stratified surface, some of them are nearly vertical, and a little of the filling surface of the backfill body will show the phenomenon of dislocation.

The failure of the delamination backfill is mostly the failure of crushing and swelling, and the lower backfill basically maintains the intact shape, while the failure of the non-delamination backfill is more longitudinal cracking, and the residual strength is greatly reduced. After the completion of loading, different degrees of separation and dislocation occur in the specimens of the delamination filling body at different levels, and the influence of the separation and dislocation caused by the Angle factor is much greater than that caused by the amount of delamination on the delamination filling body. After the interruption of filling, a low-strength interlayer is formed between the layers, which is easy to be damaged when the loading test is carried out, thus reducing the overall bearing capacity of the filling body, and leading to different degrees of separation and dislocation of the failure specimen at different layers.

## 4. Discussion

The numerical simulation of uniaxial compression of cemented tailings backfill was carried out by using PFC-2D particle flow program.

(1) Particle distribution simulation

The tailing particle gradation in the model is consistent with the real tailing particle gradation and simplified. The radius of cemented particles is set as 3.0 × 10^–4^ m, which is slightly smaller than the minimum tailings particle radius. The diameter of the sample is 50 mm and the height is 100 mm.

(2) Parameter calibration

The meso-mechanical parameters of particles in different models are the same, and only the number of cemented particles is changed [27,28], as shown in Table 4.

(3) Model construction

This simulation does not consider the influence of slurry mass concentration and curing age, and smooth joint contact is adopted for the contact between different layers of the layered backfill. The mechanical parameters of the contact model are shown in Table 7. In the simulation process, no boundary conditions are imposed around the model, and the upper and lower ends simulate the displacement control loading conditions.

(4) Analysis of crack evolution law

**Figure 16:**
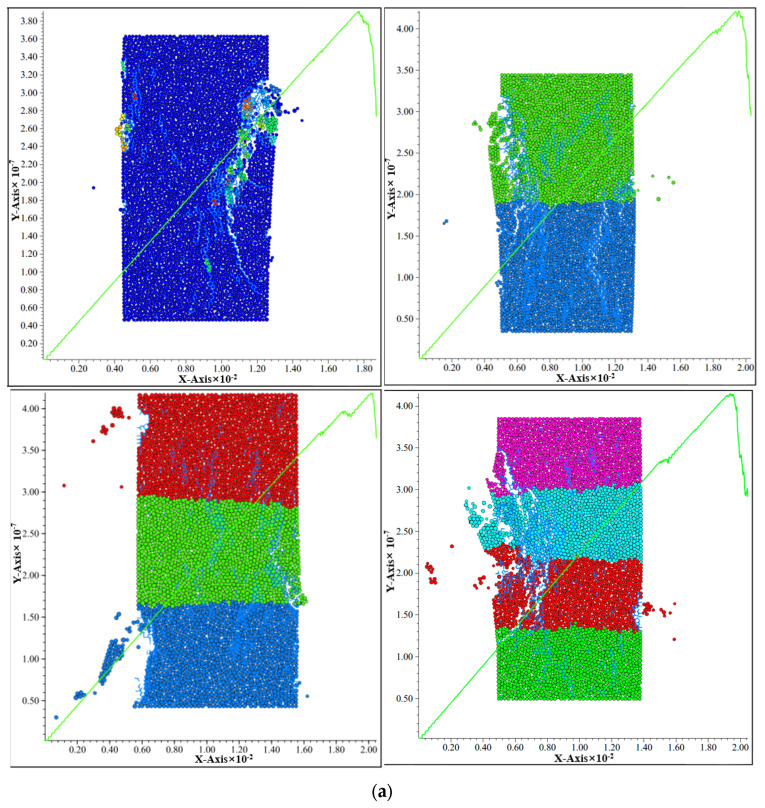

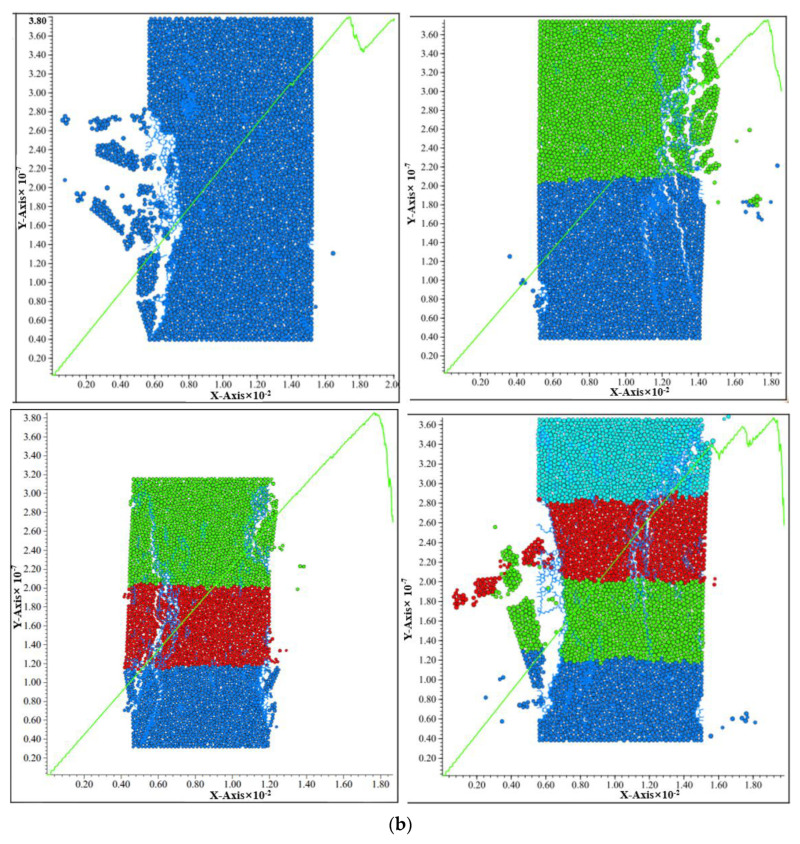

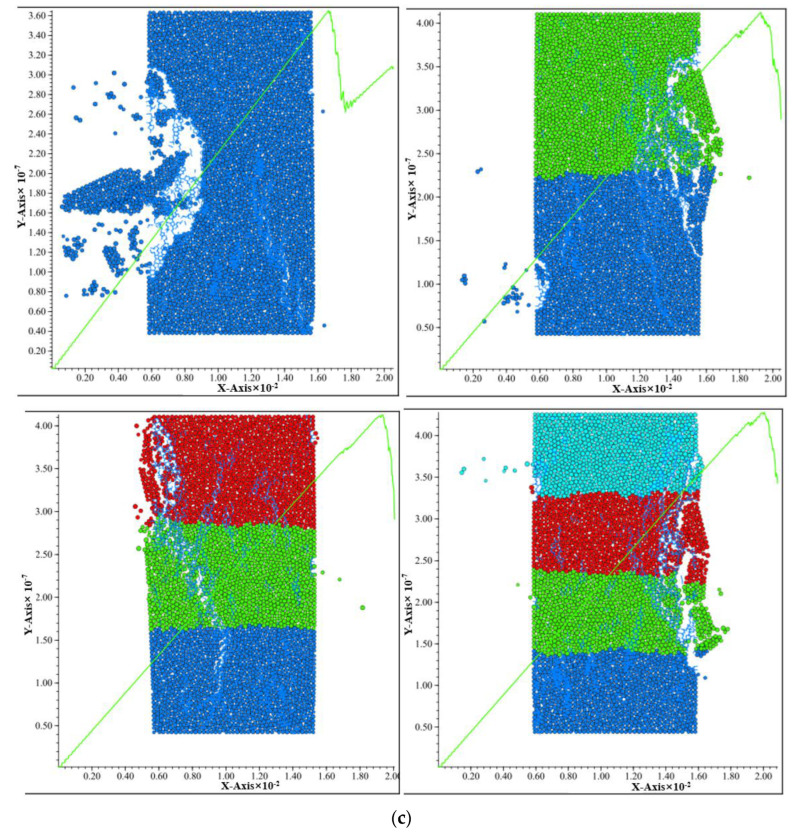
Simulation of the fracture morphology of the sample block of backfilling body: (**a**) Packing concentration 66%; (**b**) Packing concentration 68%; (**c**) Packing concentration 70%.

In essence, the failure process of backfill is the process of its internal crack initiation, expansion and connection, so it is of great significance to study the evolution law of its internal crack. The simulation results are shown in Figure 16.

At the initial stage of the stress-strain curve of the layered backfill body, the contact force between particles is less than the strength of the bond between particles, the bond between particles is not damaged, and no cracks occur in the layered backfill body. With the loading process, the contact force between particles increases gradually and begins to exceed the bond strength. The bond strength is destroyed, and internal cracks begin to occur, and the crack increment curve begins to fluctuate. When the load continued to be applied, more and more contact forces between particles exceeded the bonding bond strength, and more bonding bonds were destroyed. At this time, internal cracks began to increase, and the increment curve of cracks rose slowly. When the external load gradually reached 80% of uniaxial compressive strength, the cumulative crack curve showed inflection point and increased rapidly, and the increment crack curve also jumped. When the load continued to be applied, the stress-strain curve began to decline, the crack accumulation curve continued to grow at a high speed, and the crack increment curve remained stable at a high level. Because the two parameters of PFC2D software model, porosity and friction coefficient between particles, are parameters that cannot be obtained in laboratory tests, they can only be roughly judged based on the experience of repeated tests and previous research, so some unavoidable error values will appear. Comparing the model obtained by the software with the failure mode of the laboratory block in Figure 15, it shows the through tensile failure and half through shear failure parallel to the loading direction. The whole backfill specimen shows tensile failure, but at the same time it shows shear failure trend along the backfill surface, which indicates that the numerical simulation results of uniaxial compression of cemented tailings backfill by PFC2D particle flow program are reliable.

The specimen of layered backfill is mainly manifested in three failure modes: shear failure accompanied by secondary shear crack, tensile failure accompanied by secondary tensile crack, and conjugate shear failure accompanied by secondary tensile shear failure [29].

**Figure 17:**
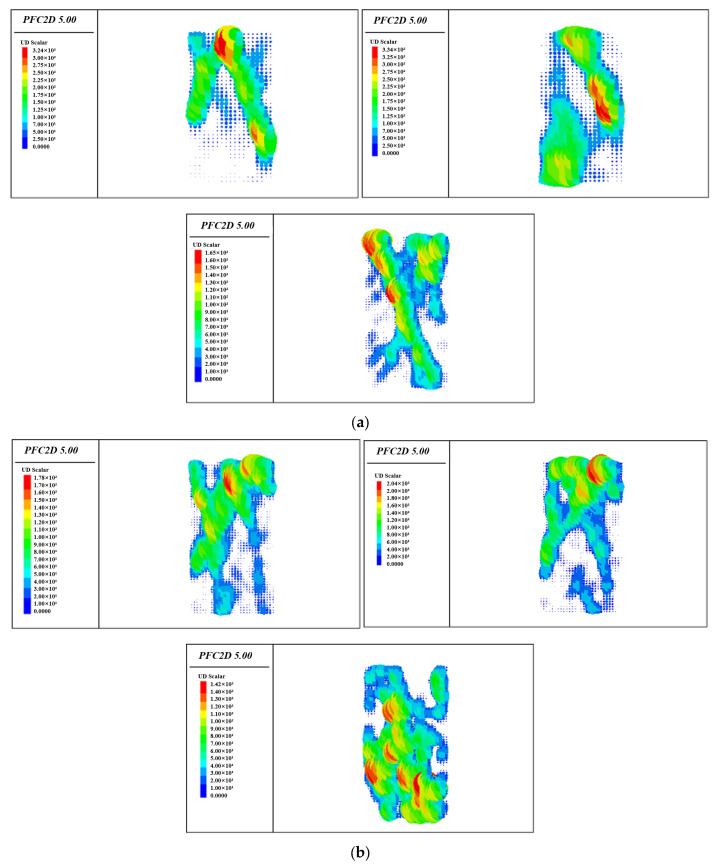

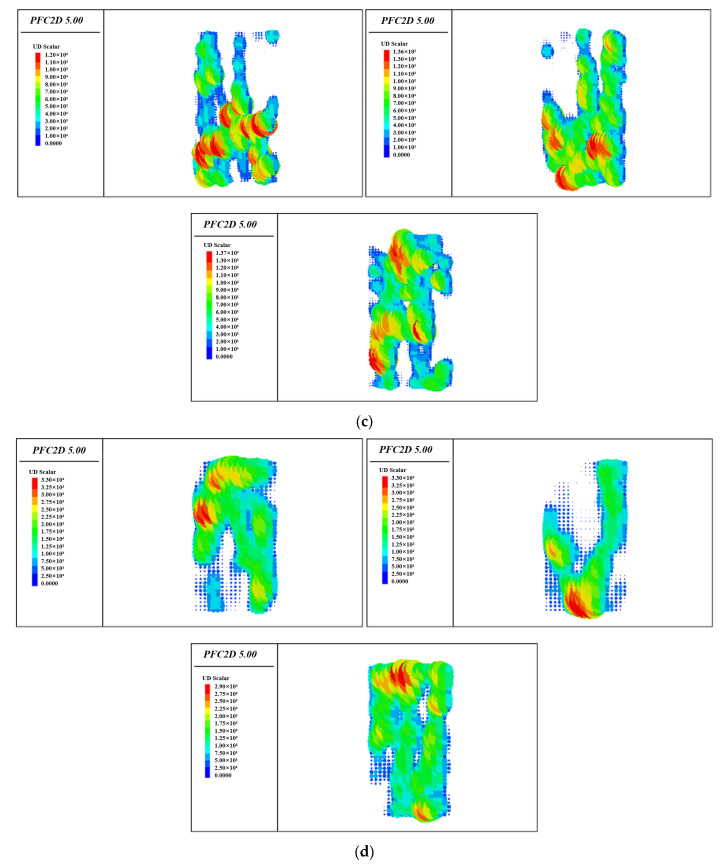
Hot spot diagram of crack distribution: (**a**) One layer; (**b**) Two layers; (**c**) Three layers; (**d**) Four layers.

According to the uniaxial compression results, the function is used to draw the hot spot map, search the segmentation number and segmentation radius. The logic is to traverse the points in the model and calculate the number of cracks within the search radius with this point as the center of the circle. Figure 17 shows the hot spots of three different mass concentrations in each layer.

The degree of crack concentration can be identified according to different colors in Figure 17. In the figure, the red area is the crack concentration area, and the blue area is the crack-free area. The hot spot map can more intuitively observe the intensity of cracks, so as to more clearly observe the damage degree of filling body.

## 5. Case Study

### 5.1. Filling Technology of Goaf

**Figure 18:**
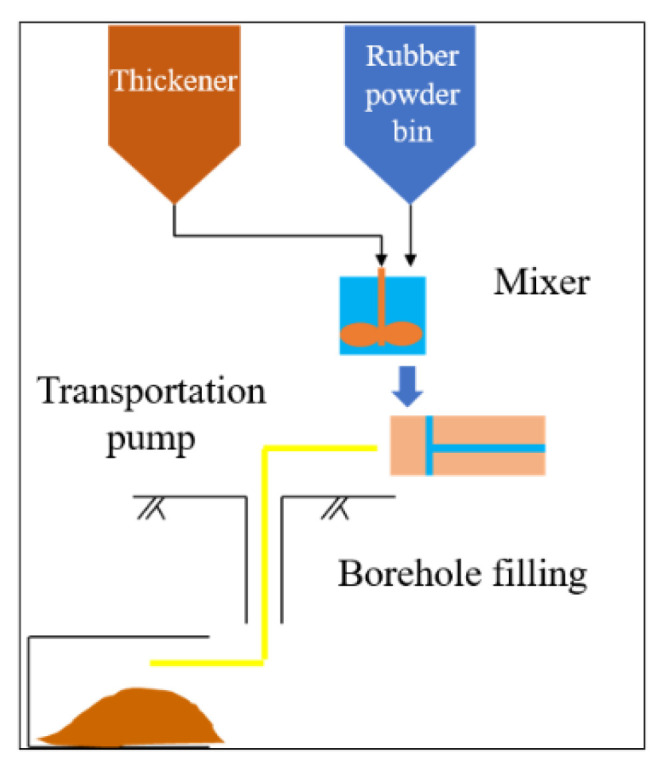
Filling process.

The filling system process of Xianglushan tungsten mine includes tailings transportation, deep cone thickening, stirring preparation, paste pumping and underground filling, as shown in Figure 18. The thickening machine has a diameter of 15 m, a total height of 18 m, and a volume of about 1800 m^3^. It is fed continuously for 24 h. When the tailings processing capacity is 1600–2400 T/d and the bottom flow concentration is 65%, the bottom flow rate is 58.18–87.27 m^3^/h, and it is directly transported to the mixing drum for filling slurry preparation. The diameter of the mixing barrel is 2000 m, and the height is 2100 mm. The ultrasonic level meter is installed on the top. The level of the mixing barrel is controlled between 1.2–1.5 m through the control system. The filled slurry stirred by two stirring drums can be switched between gravity flow and pumping through the transfer valve and conveying pipe [30,31].

Paste conveying pump two, its model is ZBJB80/8, flow 80 m^3^/h, outlet pressure 8 MPa.

1. Operation parameters of filling system:
  (1) Flow rate of filling slurry: 80–100 m^3^/h, maximum 110 m^3^/h;
  (2) Filling slurry concentration: 58–68%, maximum 72%;
  (3) Maximum filling capacity of a single system: 300–600 m^3^;
  (4) Cement–sand ratio: 1:12;
2. Key technology of downhole filling

The strength of backfill in pillar mining and normal mining is 0.5–2 MPa. The filling work of underground part is mainly to transport the prepared filling slurry in the filling station to the goaf stope that needs to be filled underground, which mainly involves the laying of filling pipe network and the construction of cofferdam retaining wall of underground stope. The cofferdam retaining wall can be filled in the stope after the completion of the above work.

### 5.2. Drainage of Filling Body in Goaf

**Figure 19:**
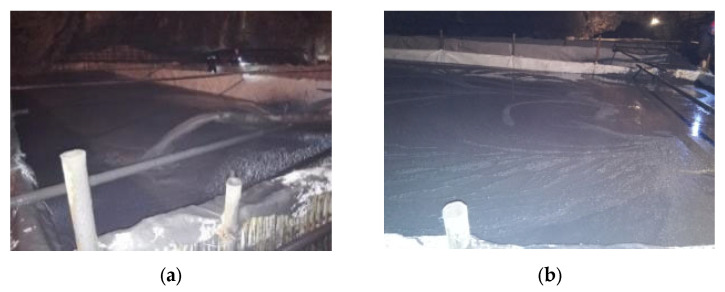
(**a**) Filling process; (**b**) Flow state.

**Figure 20:**
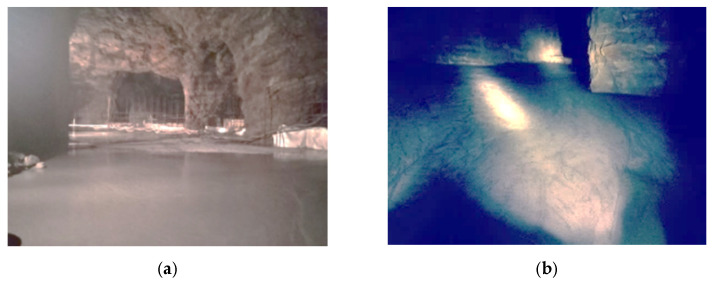
(**a**) Filter pipe arrangement; (**b**) Mining flow law in subsequent stope.

After the filling retaining wall is erected and laid, the goaf can be filled. In order to reduce the filling pressure on the filling retaining wall and control the settlement and shrinkage of the filling body, the height of a cavity filling is 0.3–0.5 m. After initial setting, the next filling is carried out with an interval of 24 h, as shown in Figure 19 and Figure 20.

Filter pipes can be arranged along the height direction of the filling goaf according to the situation, and connected to the filter pipe of the filling retaining wall to realize water filtration.

After the stope filling is completed, the requirements of upward filling mining will be met. After 28 days, when the strength of the filling body reaches the requirements of LHC rolling, the workers can drill and blast the roof on the filling body to realize effective mining of the roof ore.

In order to ensure that the filling strength meets the mining requirements, reduce the amount of cementing material and reduce the filling cost, cementing powder is used as the cementing material, and the slurry ratio of 1:12 is adopted for filling, which is between 1.5–2 MPa to meet the mining strength requirements.

### 5.3. Promotion of In Situ Downhole Curing to Backfill Strength

**Figure 21:**
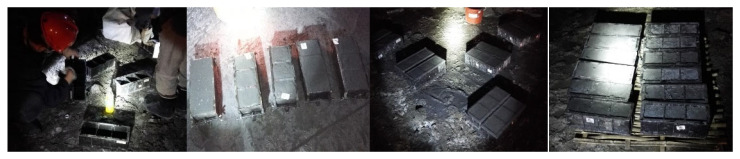
Downhole test block maintenance.

Samples were taken from the filling station and the pipe entrance of underground stope to test the strength of in situ downhole curing. The production process of downhole samples is shown in Figure 21, and the detection results are shown in Table 8.

Under the condition of the same ratio and concentration, downhole environmental conservation has a significant promoting effect on the strength of backfill, which increases from 1.81 MPa to 2.00 MPa, with a growth rate of 10.5%.

Downhole strength improvement is influenced by many factors [32,33]. The transportation time of filling slurry in different mining areas is 20–30 min. The downhole curing temperature is high and the humidity is low. The downhole temperature is 18–22 °C and the humidity is about 90%. Due to poor ventilation conditions in the filling space in the closed underground space, hydration heat cannot be dispersed during the hardening process of the filling body, resulting in a further increase in curing temperature.

**Figure 22:**
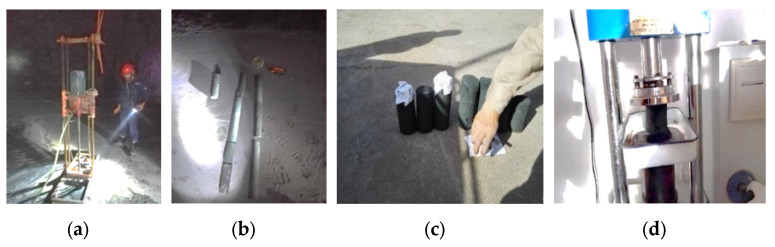
Drilling core verification of in situ backfill strength: (**a**) Drill sampling; (**b**) Sample of backfill is drilled; (**c**) The sample processing; (**d**) Mechanical property test.

The strength of in situ backfill was sampled and tested in the backfill stope. The sampling method was on-site core drilling, and uniaxial strength test was conducted after grinding. The sampling time was 28 days after filling, as shown in Figure 22.

The results of in situ downhole sampling showed that the average filling strength was 2.42 MPa, 0.61 MPa higher than the standard curing strength, with a growth rate of 33.7%, and 0.42 MPa higher than the downhole test block curing strength, with a growth rate of 21%.

The sampling intensity is greater than the target intensity, indicating that the filling process and material ratio can meet the requirements of goaf filling and residual mining, which is helpful to realize the coordination of goaf treatment and residual mining under complex conditions, and realize the safe mining of residual ore resources to the maximum while eliminating the safety hazards of goaf.

## 6. Conclusions

When the full-tail mortar concentration is 66%, the cement–sand ratio is 1:12, the filling slurry concentration is about 68%, the collapse degree is 24.2 cm, and the standard curing R28 strength of the backfill body reaches 1.86 MPa. When the filling body concentration is 66–70% and the filling times are 1–4 times, the strength reduction coefficient is 61.31–92.96%, indicating that the compressive strength weakening effect of the filling body becomes more obvious with the increase of filling times. When the filling slurry concentration is 64%, 66%, 68% and 70%, the included Angle of multiple filling layers is set as 2°, 4°, 6° and 8°, respectively, the uniaxial strength reduction ratio of the filling body at 2° is about 89–91%, the uniaxial strength reduction ratio of the filling body at 4° is about 67–74%, and the uniaxial strength reduction ratio of the filling body at 6° is about 50–68%. At 8°, the reduction ratio of uniaxial strength of backfill is about 43% and 50%. When the delamination Angle increases by 2°, the strength reduction rate of backfill stays at 20–30% on average. The greater the delamination Angle, the greater the intensity reduction factor. The downhole test block curing results show that under the condition of the same ratio and concentration, the downhole environmental curing has a significant promoting effect on the strength of backfill, which increases from 1.81 MPa to 2.00 MPa with a growth rate of 10.5%. The results of in situ downhole sampling showed that the average filling strength was 2.42 MPa, 0.61 MPa higher than the standard curing strength, with a growth rate of 33.7%, and 0.42 MPa higher than the downhole test block curing strength, with a growth rate of 21%. When the sampling depth increases from 1 m to 5 m, the strength increases from 2.26 MPa to 2.69 MPa, with a growth rate of 18.2%. The sampling depth of backfill samples represents the increase of drainage conditions and overburden pressure and other curing conditions after pouring. The greater the depth, the greater the overburden curing stress, the longer the filtration time. However, due to the limitation of conditions, the content of this experiment does not include the SEM scanning experiment of the sample, so it is impossible to further analyze the internal micro-morphology, particle size and structural defects of the sample. When the conditions permit, the author will continue the research in this direction, scan and analyze the samples by SEM, and propose a new method to determine the strength of backfill under the multi-demand of goaf treatment based on the concept of mining and backfilling.

After treatment, the amount of mined-out area and pillar is significantly reduced and the safety is significantly improved. From 2013 to 2020, the volume of mined-out area increased by mining in the eastern mining area is about 506,500 m^3^, and the volume of mined-out area eliminated by cemented filling is about 1.6738 million m^3^, and the volume of mined-out area decreases by 1.1673 million m^3^, with a reduction rate of 47.1%. The research results provide a theoretical basis for the strength problems of tailings solid waste resources in the filling process, solve the problems of solid waste resources filling, realize the effective disposal and utilization of tailings solid waste resources, reduce the amount of solid waste from the source, and promote green and sustainable development.

## Figures and Tables

**Table 1:** Single layer flow test scheme and results.

Weight Concentration	Solid Rubber Powder/kg	Tailings/kg	Water/kg	Stop Distance/cm	Stop Height/cm	Angle of Slope/°
72%	0.23	2.8	1.18	16.8	3.5	8
70%	0.23	2.7	1.25	20.9	2.9	5.5
68%	0.22	2.6	1.33	25.8	2.5	4.5
66%	0.21	2.5	1.40	31.6	2.3	3.9

**Table 2:** Particle size distribution in the first layer.

Sampling Point/(cm)		d10 (μm)	d30 (μm)	d50 (μm)	d90 (μm)
0	Underside	35.57	137.56	259.58	405.87
	Surface	0.093	0.299	1.156	215.910
10	Underside	10.07	104.95	282.98	444.16
	Surface	0.145	0.673	2.379	197.290
20	Underside	6.42	55.82	125.7	309.68
	Surface	0.111	0.562	2.603	180.280
30	Underside	0.085	38.93	114.86	215.91
	Surface	0.065	0.514	7.681	150.530

**Table 3:** The sixth layer particle size distribution.

Sampling Point/(cm)		d10 (μm)	d30 (μm)	d50 (μm)	d90 (μm)
0	Underside	73.179	137.560	197.290	259.580
	Surface	0.429	3.118	12.057	156.360
10	Underside	87.64	150.53	215.91	236.28
	Surface	0.358	2.379	9.199	140.440
20	Underside	104.95	125.7	180.28	210.11
	Surface	0.327	2.174	8.406	137.560
30	Underside	95.91	114.86	164.74	197.29
	Surface	0.429	1.986	7.681	133.240
40	Underside	87.640	104.95	146.23	177.22
	Surface	0.469	1.815	7.019	130.110
50	Underside	35.573	95.91	140.17	160.14
	Surface	0.228	1.659	3.412	129.070
60	Underside	38.93	55.836	90.12	155.29
	Surface	0.209	1.266	2.850	127.370
70	Underside	0.358	5.861	9.12	130.22
	Surface	0.191	1.156	2.603	127.870

**Table 4:** 28-day strength of slicing backfill.

Filling Concentration	Uniaxial Compressive Strength at Different Filling Times/MPa			
	1st	2nd	3rd	4th
70%	1.99	1.59	1.51	1.39
68%	1.85	1.51	1.34	1.23
66%	1.78	1.41	1.37	1.22

**Table 5:** Strength reduction coefficient results of backfill.

	1	2	3	4
70%	100.00%	79.90%	75.88%	69.85%
68%	92.96%	75.88%	67.34%	61.81%
66%	89.45%	70.85%	68.84%	61.31%

**Table 6:** Reduction coefficient of uniaxial compressive strength of inclined slicing backfill.

Mass Concentration	0°	2°	4°	6°	8°
70%	100.00%	89.42%	74.43%	68.09%	50.56%
68%	100.00%	91.74%	76.47%	64.08%	43.82%
66%	100.00%	90.71%	76.43%	67.29%	43.78%
64%	100.00%	89.08%	67.43%	50.09%	44.16%

**Table 7:** Meso-mechanical parameters of numerical models.

Type	Parameter	Value
Tailings particles	Density/(kg·m^−3^)	2700
	Porosity	0.4
	Fric	0.5
	Kn/(N·m^−1^)	6.0 × 109
	Ks/(N·m^−1^)	6.0 × 109
	Radii of particles/m	4.1 × 10^−4^ − 3.0 × 10^−3^
Cement particles	Density/(kg·m^−3^)	3200
	Porosity	0.5
	Fric	0.5
	Kn/(N·m^−1^)	6.0 × 109
	Ks/(N·m^−1^)	6.0 × 109
	Radii of particles/m	3.0 × 10^−4^
Parallel bond contact	Pb_emod/(N·m^−1^)	1.0 × 109
	Pb_coh/(N·m^−1^)	4.0 × 108
	Pb_ten/(N·m^−1^)	2.0 × 108
	Pb_radius	1
Smooth joint contact	sj_Kn/(N·m^−1^)	200 × 109
	sj_Ks/(N·m^−1^)	200 × 109
	sj_fric	0.1
	sj-large	1

**Table 8:** Comparison of subsurface maintenance results.

Place	Proportion	Concentration/%	Strength/MPa	Average Strength/MPa	Days
Filling station	1:12	68	0.53/0.48/0.51	0.36	3
Under pit	1:12	68	1.1/0.7/1.1/0.4/0.5/0.3	0.68	3
Filling station	1:12	68	0.95/0.78/0.82	0.61	7
Under pit	1:12	68	1.4/1.3/0.6/0.7/1.4/1.7/0.8/0.4	0.85	7
Filling station	1:12	68	1.84/1.83/1.78	1.81	28
Under pit	1:12	68	2.5/2.5/1.3/2.8/2.5/2.7/3.1/1.2/	2.00	28

## Data Availability

Data are contained within the article.
